# New metric for optimizing Continuous Loop Averaging Deconvolution (CLAD) sequences under the 1/f noise model

**DOI:** 10.1371/journal.pone.0175354

**Published:** 2017-04-17

**Authors:** Xian Peng, Han Yuan, Wufan Chen, Tao Wang, Lei Ding

**Affiliations:** 1 School of Biomedical Engineering, Southern Medical University, Guangzhou, Guangdong, PR. China; 2 Stephenson School of Biomedical Engineering, University of Oklahoma, Norman, Oklahoma, United States of America; Duke University, UNITED STATES

## Abstract

Continuous loop averaging deconvolution (CLAD) is one of the proven methods for recovering transient auditory evoked potentials (AEPs) in rapid stimulation paradigms, which requires an elaborated stimulus sequence design to attenuate impacts from noise in data. The present study aimed to develop a new metric in gauging a CLAD sequence in terms of noise gain factor (NGF), which has been proposed previously but with less effectiveness in the presence of pink (1/f) noise. We derived the new metric by explicitly introducing the 1/f model into the proposed time-continuous sequence. We selected several representative CLAD sequences to test their noise property on typical EEG recordings, as well as on five real CLAD electroencephalogram (EEG) recordings to retrieve the middle latency responses. We also demonstrated the merit of the new metric in generating and quantifying optimized sequences using a classic genetic algorithm. The new metric shows evident improvements in measuring actual noise gains at different frequencies, and better performance than the original NGF in various aspects. The new metric is a generalized NGF measurement that can better quantify the performance of a CLAD sequence, and provide a more efficient mean of generating CLAD sequences via the incorporation with optimization algorithms. The present study can facilitate the specific application of CLAD paradigm with desired sequences in the clinic.

## Introduction

Repetitive short sound stimuli are usually employed to elicit transient auditory evoked potentials (AEPs) reflecting time-deterministic and phase-locked electrophysiological activities along the auditory pathway [1]. These electrophysiological responses have important clinical applications in diagnosis of hearing problems [2] and configuring hearing assistive devices [3]. Traditionally, stimulus repetition rate must be low enough to avoid overlap of electrophysiological responses to consecutive stimuli. For example, the earliest AEP component recorded over the scalp, termed as auditory brainstem response (ABR), occurs at a latency approximately between 1.5 ms and 10 ms after stimulus onset. Conventional averaging method for ABR requires that stimulus onset asynchrony (SOA) must be adequately larger than 10 ms, corresponding to a maximum of 100 Hz stimulation rate. In the case of middle latency responses (MLRs), which present even much later, the stimulation rate should be limited to 12 Hz or lower.

Rapid stimulation elicits periodical responses termed as steady state response (SSR) with a corresponding SOA [4]. In particular, the stimulation at the rate of 40 Hz has been widely-used in audiometry for greatly enhanced amplitude in responses. SSR has been well explained in a superimposition theory, which indicates that overlapped transient AEPs account for the generation of SSR [5,6]. The overlapping is mathematically equivalent to a convolution process between the stimulus sequence and transient AEP response to individual stimuli. Therefore, many deconvolution algorithms have been developed to recover transient AEPs from overlapped responses, i.e., SSR. In order for deconvolution algorithms to work, stimulation sequences should not be evenly spaced. Rather, jittered-sequences with varying SOAs have been designed for most deconvolution algorithms. The maximum length sequence (MLS) derived using the primary polynomials presents desired properties that allow rapid and stable solutions of inverse problem from the convolution model [7]. For a given MLS, there is an associated inverse sequence that can be used to directly reconstruct convolved variables in the time domain via operating a correlation analysis. However, a MLS generated using a particular primary polynomial consists of a set of pseudo-random binary sequences with a few SOA values of integral multiples, implying large stimulus jitters in the sequence. Large sequence jitters may deteriorate the prerequisite of linear convolution model in transient AEPs and SSRs due to adaptation effects. Another concern using MLS is its inflexible configuration of number of stimuli in a sweep that increase exponentially with the order of MLS. In the domain of event-related potentials, Woldorff (1993) has developed a series of algorithms in the time domain to deconvolve overlapped adjacent responses, which are collectively referred as the “Adjar” (Adjacent response) procedure [8]. The ADJAR is relatively difficult to implement and computationally expensive. It also requires wide range of SOA jitters to remove overlaps from low-frequency components of evoked responses [9].

Small and flexible jitter sequences allowing the development of algorithms for efficient transient AEP recoveries are ideal for rapid stimulation paradigms. One line of research towards this goal has been the development of continuous loop averaging deconvolution (CLAD)[10,11], or q-sequence deconvolution methods [12]. The CLAD method uses random SOAs with relatively low and arbitrary jitter to recover transient AEPs. It is usually implemented in the frequency domain to take the computational advantage of fast Fourier transform (FFT). To date, multiple related techniques have been introduced, leading to several new deconvolution algorithms, such as the randomized stimulation and averaging method proposed by Valderrama et al [13], least-squares deconvolution method by Bardy et al [14], and multi-rate steady-state averaging deconvolution method by Wang et al [15]. These methods have been found valuable for studies of transient AEPs and their roles in the generation of SSRs [16–18]. They have also been used in investigating basic neuroscience problems, e.g., neuronal adaptation phenomena observed from high-rate ABRs [19] and ABRs simultaneously with electrocochleography [20], and in assisting clinical procedures, e.g., anesthesia monitoring [21,22].

Among these methods, the CLAD method addresses the problem straightforwardly in the frequency domain with unbiased estimation and perfect reconstruction on noise-free condition. One of common efforts for implementing CLAD is its design and evaluation of stimulus sequence [11,23]. Theoretically, the SOAs for a CLAD sequence could follow an arbitrary distribution within a predefined range in the noise-free context. However, the performance of deconvolution is highly dependent on stimulus sequences when noise is present in signals. This is because sequence-dependent deconvolution filters can amplify noises/artifacts in recordings at certain frequencies, introducing distortions to AEPs to be recovered.

The noise gain property of deconvolution methods thus offers an objective measure for evaluating the goodness of stimulus sequences. Özdamar and Bohórquez[11] proposed a noise gain factor (NGF) called *C*_dec_, to measure noise amplification or attenuation in the process of inverse filtering for signal deconvolution. However, this factor weighs contributions over frequencies uniformly, which is only suitable for white noise. It is well known that spontaneous EEG, appearing as noise on transient AEPs, often follows the power-law distribution in the frequency domain. Such a violation upon the assumption may impact the usefulness of the metric *C*_dec_ [11]. In the present study, we examined the effects of using *C*_dec_ on stimulus sequence selection and on recovering transient signals in the context of noise with the power-law spectrum. We then developed a novel NGF metric (denoted as *G*_dec_) with the consideration of the power-law spectral distribution of spontaneous EEG noise. Finally, we compared the performance of both metrics (*C*_dec_ vs. *G*_dec_) on sequence optimization and selection, as well as in reconstructing transient AEPs using real EEG data.

## Materials and methods

### CLAD method and Noise Gain Factors (NGFs)

**Fig 1:**
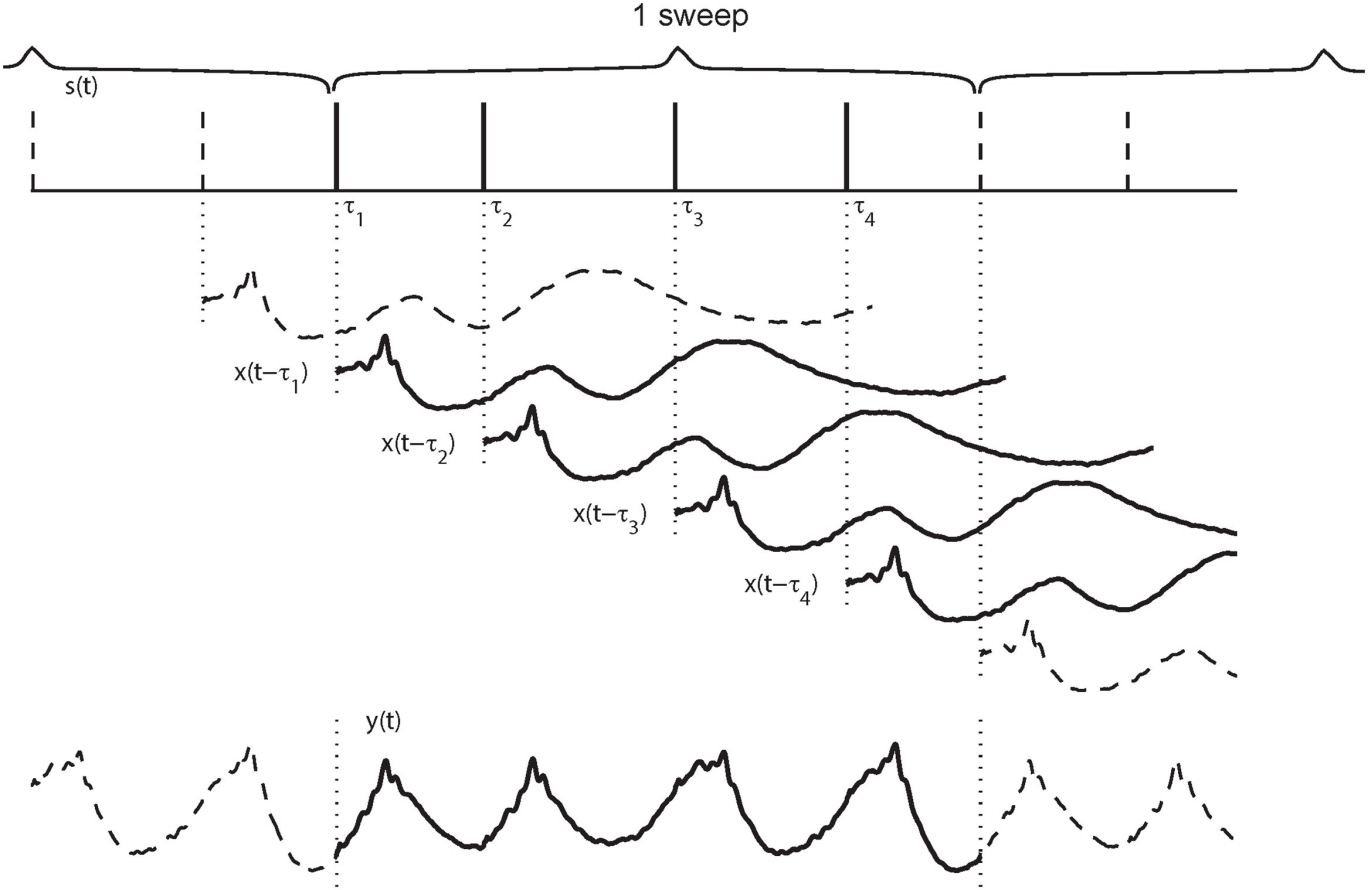
Diagram of convolved response. The stimulus sequences *s*(*t*) containing four short SOAs, is presented repeatedly, i.e., in *s*_*T*_(*t*). Responses to individual stimuli, *x*(*t*), with lags (*τ*_*i*_) corresponding to the SOAs are superimposed to produce a sweep (period) of the convolved (overlapping) response *y*(*t*).

In the CLAD method, a stimulus sequence is periodically delivered to evoke brain responses. The stimulus sequence typically contains several individual stimuli with varying SOA. The use of varying SOA is termed as the jittering technique. Jitters disrupt periodical responses within the stimulus sweep provided that SOAs are shorter than transient responses to individual stimuli. This response is assumed to be unitary regardless the SOA variation. In this case, the observed response can be modeled as a convolution between the transient response and the stimulus sequence as shown in Fig 1. Since the stimulation sweep is presented in the manner of continuous loop so that averaging over sweeps can be utilized to enhance signal-to-noise ratio in resulted responses. Mathematically, the resulted response *y*(*t*) due to a stimulus sweep *s*(*t*) in the presence of additive noise *e*(*t*) is expressed as:

$$y(t)=s(t)⊗x(t)+e(t)=\int_{0}^{T}s(\tau )x(t-\tau )d\tau +e(t),t\in [0,T]$$ (1)

where *x*(*t*) is the supposed transient response to individual stimuli, the symbol ⊗ denotes the circular convolution operator due to looped stimulation, and *T* denotes the length of the stimulus sweep. (Eq 1) represents a continuous convolution in the time domain. The corresponding version in the frequency domain is denoted in the equation below using capital letters

Y(f)=S(f)X(f)+E(f),f∈[−∞,∞] (2)

which yields the estimated solution of

$$\hat{X}(f)=Y(f)S^{-1}(f)+E(f)S^{-1}(f)$$ (3)

Two prerequisites are necessary for (Eq 3) to have unique and reliable solutions. First, the inverse filter can be obtained uniquely, which is defined as a reciprocal of the stimulus sequence in the frequency domain. Obviously, the nontrivial solution is mathematically impossible for an isochronic-SOA sequence even in noise-free conditions [12]. The CLAD method thus depends on jittered sequence to address the ill-posed condition in (3). Second, the inverse filter should not introduce large distortions when filtering noise, *E*(*f*). Appropriate jittering techniques should be used to minimize the impact of inverse filtering of noise on deconvolution results. Otherwise, if *S*(*f*) approaches zero at some frequencies, the inverse filter *S*^−1^(*f*) is going to over-amplify noise *E*(*f*) corresponding to those frequencies. It is therefore ideal that the amplitude spectrum of the inverse filter, |*F*(*f*)|, should be small and flat within the frequency range, *f* ∈[*f*_*L*_, *f*_*H*_] Hz, of AEPs to be recovered.

The time-discrete stimulus sequence can be expressed as follows:

$$s[n\Delta t]=\left\{ \begin{array}{l} 1,\begin{array}{cc} & \text{stimulus}\begin{array}{c} \text{onset} \end{array} \end{array}\\ 0\begin{array}{ccc} & & \text{no}\begin{array}{c} \text{stimulus} \end{array} \end{array} \end{array}\right.$$ (4)

where [·] denotes a digital variable, Δ*t* denotes the discretized time interval. The stimulus sequence is considered as a binary sequence in which an "1" indicates the onset of a stimulus.

The deconvolution in (Eq 3) can be achieved through the FFT algorithm [11,12]. The inverse filter is obtained from the discrete FFT, ℱ{⋅}, on this stimulus sequence:

$$S[k\Delta f]=ℱ\{s[n\Delta t]\}=\sum_{n=1}^{N}s[n\Delta t]e^{-\text{j}\frac{2\pi }{N}kn},k=1,2,\ldots ,N.$$ (5)

Specifically, *F*[*k*Δ*f*] = 1/*S*[*k*Δ*f*], where Δ*f* = 1/(*N*Δ*t*) = 1/*T* represents the frequency interval or the frequency resolution that is determined by the length of stimulus sweep *T*. The valid frequency band is represented with discrete frequency indices as, and.

Within the frequency band [*k*_L_, *k*_H_], the amplitude of *F* contributes unevenly in amplifying the noise term in (Eq 3). To quantify the noise amplification associated with a stimulus sequence, the NGF is defined as

$$C_{\text{dec}}=\sqrt{\frac{1}{k_{\text{H}}-k_{\text{L}}}\sum_{k=k_{\text{L}}}^{k=k_{\text{H}}}|F[k\Delta f]|{}^{2}}$$ (6)

Essentially, *C*_dec_ is the total gain for all bins within the frequency band. Smaller *C*_dec_ indicates smaller noise artifacts introduced from stimulus-sequence-dependent inverse filtering. When *C*_dec_ < 1, noise is attenuated.

### Fourier transform for time-continuous CLAD sequence

Although the classical discrete CLAD based on FFT is suitable in digital computation, the time-discrete sequences are inconvenient to be applied to different acquisition systems. A transfer of discretized-rate of a sequence may change its designated frequency property to certain degree due to the modification of SOAs. In addition, discrete sequences also limit its searching space when optimization algorithms are applied to identify desired sequences. To develop a time-continuous sequence for CLAD computation, a sweep of stimulus sequence that contains *P* individual stimulus occurrences can be considered as a time-delayed summation of delta function,

$$s(t)=\sum_{p=1}^{P}\delta (t-\tau_{p}),$$ (7)

where the time delay variable *τ*_*p*_, *p* = 1,2,…,*P*, indicates time points of stimulus occurrences. The interval between two consecutive occurrences Δ*τ*_*p*_ = *τ*_*p*_ − *τ*_*p*−1_, varies, leading to sequence jitters.

We define a jitter ratio (JR) to describe the irregularity of SOA in a sequence,

$$JR=\frac{\text{max}(\Delta \tau_{p})-\text{min}(\Delta \tau_{p})}{\bar{\tau }},$$ (8)

where is the mean-SOA of the sequence. *JR* represents the ratio between the maximum SOA difference and the mean-SOA of a stimulus sequence. For most applications, small JR is preferred as it offers pseudo-identical stimulation condition for each stimulus occurrence.

In CLAD experiments, the stimulus sequence is repetitively presented with a period of *T*, the entire stimulus session is a periodic extension of *s*(*t*) (Fig 1). The total stimulus train can be expressed as:

$$s_{T}(t)=s(t)⊗\delta_{T}(t-qT),$$ (9)

where *δ*_*T*_(*t*) is a periodic impulse function with period *T* defined as:

$$\delta_{\text{T}}(t)=\sum_{q=-\infty }^{\infty }\delta (t-qT).$$ (10)

The Fourier transform of is an infinite discrete sequence modulated collectively by a number of exponential functions with different frequencies, i.e,

$$S[k\Delta f]=ℱ\{s_{\text{T}}(t)\}=2\pi \Delta f\sum_{p=1}^{P}e^{-\text{j}k2\pi \Delta ft_{p}},k\in [-\infty ,\infty ].$$ (11)

Hence, the inverse filter becomes *F*[*k*Δ*f*] = 1/*S*_T_(*k*Δ*f*), where Δ*f* = 1*/T* is analogous to the one defined in the discrete format and represents the frequency resolution. Note that, unlike the periodical discrete-time filter, the continuous-time inverse filter is of infinite length in the frequency domain, but the values outside the frequency range defined by time-discrete have no effect on the deconvolution calculation. (Eq 11) also implies that using different discrete rates is viable for the stimulus sequence, on the condition that it is supported by the simulation system, because the frequency resolution is only determined by the sweep length *T*.

### New NGF metric for 1/f EEG noise

The NGF defined in (Eq 6) consider noise contribution over the chosen frequency band of equal weights, which is equivalent to an assumption of a white noise condition. However, the spectrum of background EEG is far from white in practice, which are contributed by various source origins. Spontaneous activity of cortical neurons and scalp muscle activity account for a large amount of these background activities. Electromagnetic interference and electrical noise in the acquisition system also represent a notable proportion. The frequency distribution of combined background EEG signals and noise can be accurately described as a color noise or (*α* ≥ 0) process [24], such that its power spectral density is inversely proportional to its frequency. Considering that its power spectrum is of 1/*f*^*α*^ distribution, the original *C*_dec_ is not appropriate with uniform weights for all frequency bins within considered frequency band.

To account for actual background EEG power spectrum, we modify the NGF at first in time domain as a ratio between noise after inverse filtering, denoted *e*_*f*_(*t*), and pre-deconvolved noise, *e*(*t*), in terms of their root mean square (RMS) values:

$$G_{dec}=\frac{\text{rms}(e_{f})}{\text{rms}(e)}=\frac{\sqrt{\sum e_{f}^{2}(t)}}{\sqrt{\sum e^{2}(t)}}$$ (12)

Rewriting (Eq 12) in the frequency domain and considering only the necessary frequency range

$$G_{\text{dec}}=\frac{\sqrt{\sum_{k=k_{\text{L}}}^{k=k_{\text{H}}}|E(k\Delta f)F_{\text{T}}(k\Delta f)|{}^{2}}}{\sqrt{\sum_{k=k_{\text{L}}}^{k=k_{\text{H}}}|E(k\Delta f)|{}^{2}}}$$ (13)

Since the *e*(*t*) is assumed to be of a 1/*f* noise, i.e., $\left|E(k\Delta f)\propto 1/f^{\alpha }\right|$, (Eq 13) becomes

$$G_{dec}=\frac{\sqrt{\sum_{k=k_{L}}^{k=k_{H}}\frac{1}{k^{2\alpha }}|F_{T}(k\Delta f)|{}^{2}}}{\sqrt{\sum_{k=k_{L}}^{k=k_{H}}\frac{1}{k^{2\alpha }}}},$$ (14)

or in a simplified form:

$$G_{\text{dec}}^{2}=\gamma \sum_{k=k_{\text{L}}}^{k_{\text{H}}}k^{-2\alpha }|F_{\text{T}}(k\Delta f)|{}^{2}$$ (15)

where $\gamma =1/\sum_{k=K_{\text{L}}}^{K_{\text{H}}}k^{-2\alpha }$is a normalizing constant so that *G*_dec_ = 1 represents neither amplification nor attenuation within the considered frequency band.

In this equation, the weights derived from the deconvolution filter, *F*_T_(Δ*f*) are adjusted by 1/*k*^*α*^, which defines a damping effect on the weight of noise gain with increasing frequency so that the magnitude at high frequency bins contributes less to the value of *G*_dec_. Comparing the equations for *C*_dec_ and *G*_dec_, it is not difficult to find that *C*_dec_ is essentially a special case of *G*_dec_ when *α* = 0.

### EEG data and parameters

The CLAD method can be applied to other transient responses than AEPs, for example, in extracting transient patterns in electroretinogram data [25], or in simultaneously recorded ABR and electrocochleography data [20]. Most applications of CLAD in AEPs are in the study of ABR and MLR recordings. In the present study, we used archive EEG data acquired previously in our lab. The EEG recordings were collected from three silver/silver chloride electrodes (Fz: positive input; right mastoid: negative input; Fpz: ground). The sampling rate for all dataset was 20 kHz (SynAmps2, Compumedics, Victoria, Australia). The analogue filter in the amplifier was set to 100–3000 Hz for ABR recordings, and 5–500 Hz for MLR recording. Acquired EEGs for MLR underwent a Butterworth digital filter of 10–350 Hz for exemplar sequences using actual CLAD paradigm.

To validate the proposed NGF measurement, *G*_dec_, and compare its performance to *C*_dec_ in the topic of stimulus sequence selection, we used a published sequence for the CLAD method in the literature [26] as an initial sequence, specifically these eight SOAs = {27.2; 36.8; 36.8; 20.8; 32.0; 19.2; 16.0; 16.0 ms}, and derived all possible sequences by performing permutation on it (i.e., randomly changing the order of these eight SOAs in the sequence). The stimulus rate was 40 Hz, as determined by the mean SOAs. From all the sequences we selected 15 sequences according to the proposed NGF metric, which represented cases from low to high noise amplification properties. These sequences were used to test background EEGs recorded for both conditions of ABR and MLR.

In the last part of the study, we further selected three exemplar sequences to reconstruct transient MLR using the CLAD paradigm at a rate of 40 Hz. Because the existence of AEP and its major components elicited at this rate have been well established[13,14,18], the purpose of this experiment is to demonstrate possible distortions on AEP caused by different sequences. The rarefaction clicks of duration of 0.1 ms were delivered monaurally to the right ear at 82 dB SPL via an insert earphone (ER-3A Etymotic Research, Elk Grove Village, IL). The sound pressure level was calibrated with a HA-2 coupler (Bruel & Kjaer DB-0138), attached to a microphone (Bruel & KJaer Type 4144) and sound level meter (Quest 1800). Artifact rejection was set at 40 μV. Around 1800 sweeps were acquired for each sequence.

We recruited five healthy adults (age 25–31, one female) with no self-reported history of auditory or neurological disorders, and ran conventional MLR tests with recording parameters as described above. They all gave their written informed consents. The study was approved by the Ethical Committee of the Southern Medical University.

### Power law spectra of spontaneous EEG data

**Fig 2:**
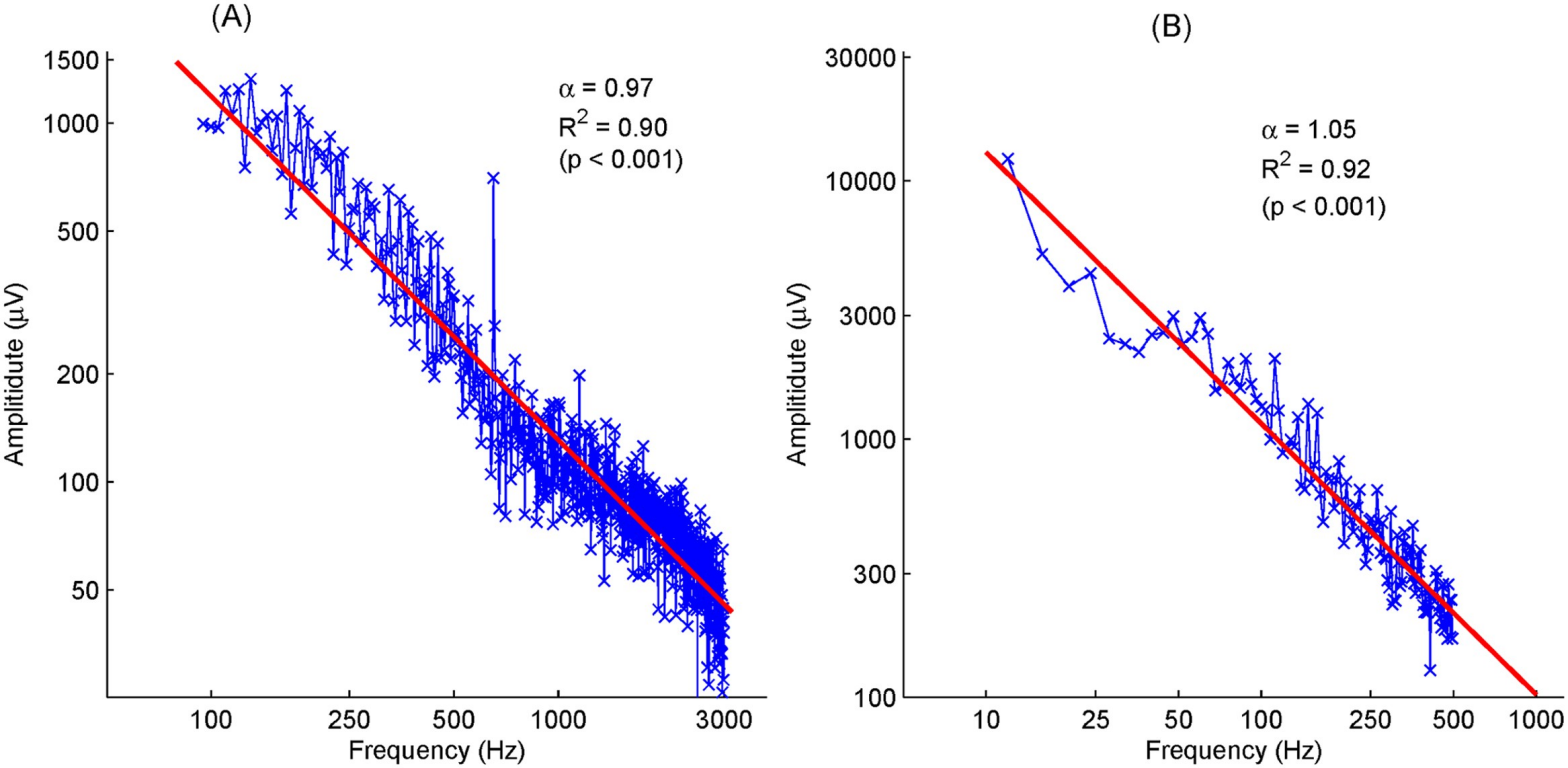
Stimulus-free EEG spectra averaged over 20 sweeps for ABR within 100–3000 Hz in (A), and for MLR within 10–500 Hz in (B), both of which represent clear 1/*f*^*α*^ scaling. The straight lines are the least-square fitting on these data, where the values of *α* and *R*^2^ are given as well.

In order to understand the noise property in typical AEPs, we analyzed spontaneous EEG data obtained from our previous studies on ABR and MLR, in which 20 randomly selected EEG epochs were used from ABR and MLR recordings, respectively. The spectra from these selected epochs were averaged to represent the typical frequency characteristics of EEG at both recording cases. The valid frequency band is 100–3000 Hz for ABR, and 10–500 Hz for MLR recordings. Their spectra within these frequency bands were presented in a logarithmic scale in Fig 2. The least-square fitting to the data demonstrate a power-law distribution of EEG data from both ABR (Fig 2A) and MLR (Fig 2B) recordings. Therefore, a 1/*f*^*α*^ scaling can be used to model EEG frequency characteristics, where the α value controls the decay rate. EEGs with smaller α values are arguably of more deteriorated signals close to white noise. For the data being analyzed, α values approach 1 and the corresponding R-squared (i.e., the coefficient of determination) approach 0.9 for both cases (*p* < 0.001), indicating a standard 1/*f* scaling of great confidence. Due to the 1/*f* distribution, we proposed *G*_dec_ over *C*_dec_, which was designed with the consideration of white noise, while *G*_dec_ considers the realistic EEG spectrum distribution (i.e., 1/*f*).

## Results

### CLAD sequences and their NGF properties

For a CLAD sequence, the SOAs and their order in the sequence define their frequency characteristics. Sequences with same SOAs exhibit same stimulation rate and JR, which are generally determined by specific applications of the paradigm. Here we investigated the effect of SOA ordering on the noise gain property of a sequence. We derived all possible sequences using same SOAs from the initial sequence which has proved to be applicable in literature [16,26]. The eight SOAs from the initial sequence were permuted to form all possible stimulus sequences. After removing these time-reversed pairs, circularly shifted sequences and redundant sequences due to two repeated SOAs (i.e., 16.0 ms and 36.8 ms), we obtained a total of 630 derived sequences with unique frequency property for analysis.

**Fig 3:**
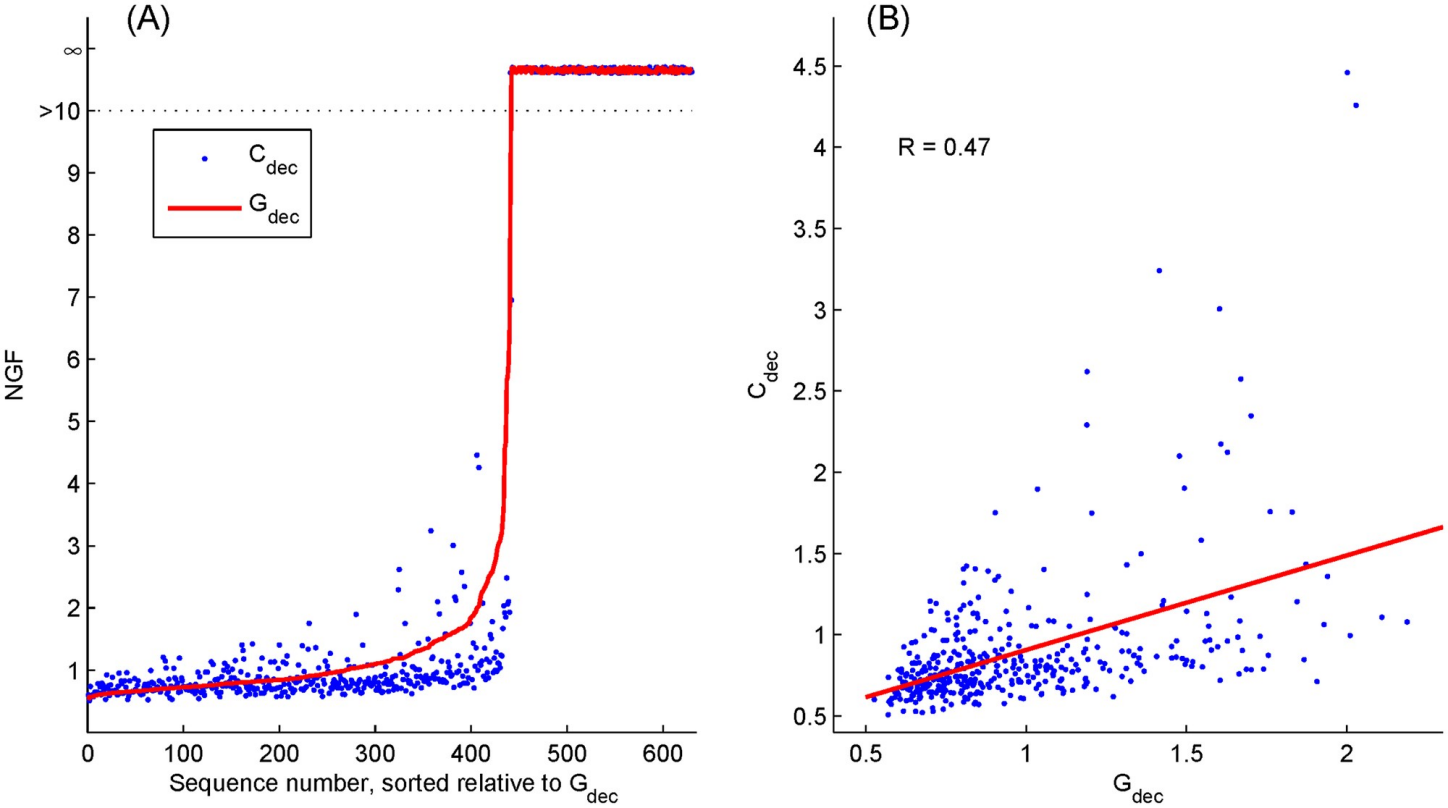
*G*_dec_ **and** *C*_dec_ **values in the frequency range of 10–350 Hz for the total 630 sequences sorted by increased** *G*_dec_ **(A). The first 410 sequences with reasonable NGFs (< 10) are selected and plotted in a scatter graph in (B), where the Pearson correlation coefficient between** *G*_dec_ **and** *C*_dec_ **is R = 0.47. **

The *C*_dec_ and *G*_dec_ of each sequence were calculated according to Eqs (6) and (14), respectively. Fig 3A shows these *G*_dec_ values sorted in an ascending order, and their corresponding *C*_dec_ values. It shows that some NGF values are dramatically large that approach infinity (see the area with condensed scales for NGF > 10). These unusually extreme values exist due to the singularity of the deconvolution process. The sequences generating these extremes are inappropriate to be used as efficient CLAD sequences in reconstructing transient evoked responses under high-rate stimulation paradigms. These extremes may occur at any frequency and, therefore, wider frequency bands usually exhibit higher chances in having extremes. In the present study, more restrictive frequency bands were used for MLR, which was 10–350 Hz (Fig 3), in order to reduce the chance of running into the singularity problem.

After removing sequences with extreme values, 410 sequences indicate that their *C*_dec_ and *G*_dec_ values are smaller than 10. The scatter plot for *C*_dec_ versus *G*_dec_ is shown in Fig 3B. The Pearson correlation coefficient between *C*_dec_ and *G*_dec_ is 0.47 (*p* < 0.001), indicating a moderate relationship among them.

**Fig 4:**
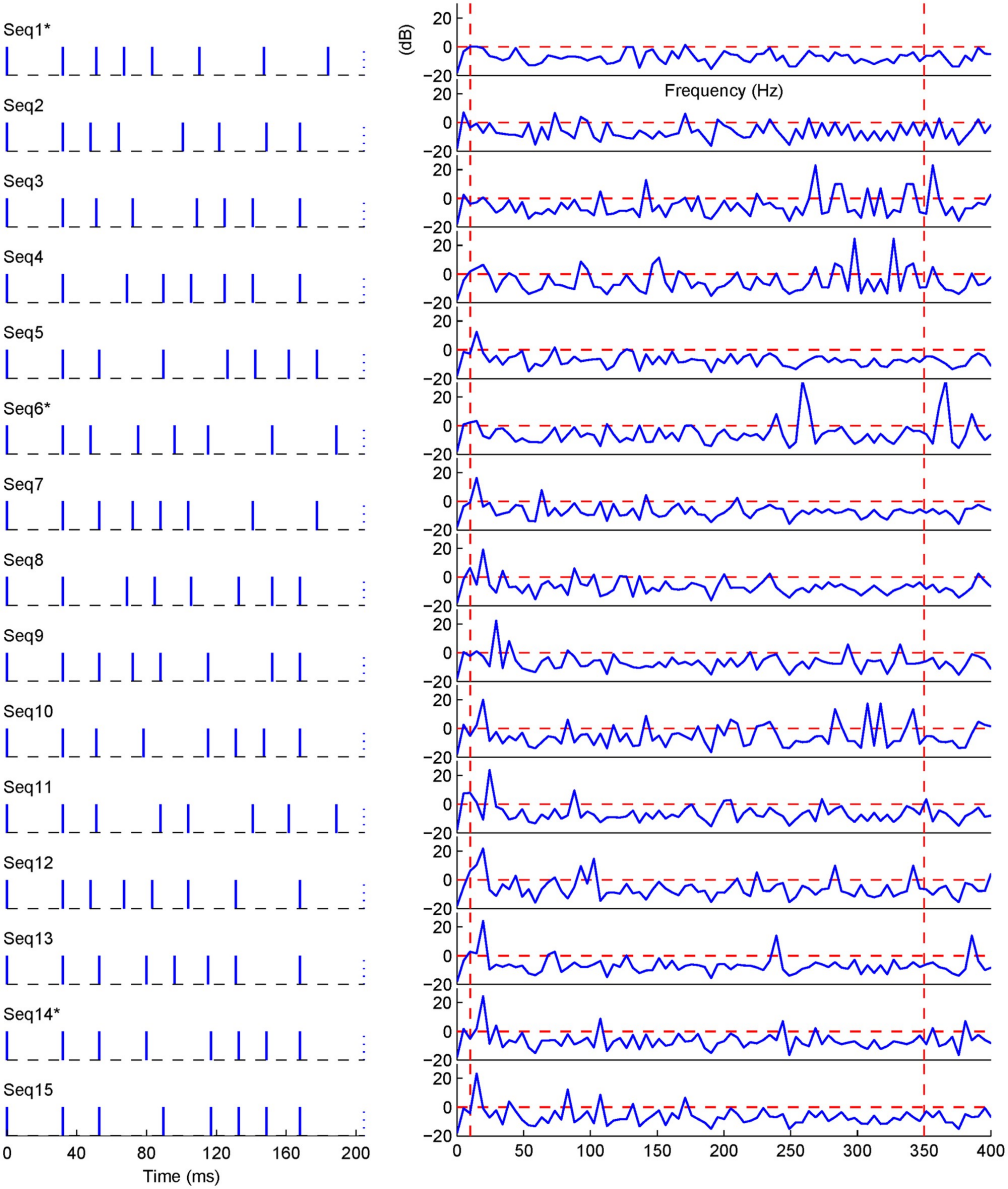
Fifteen representative sequences from Seq1 to Seq15 (left column) are shown according to ascending values of *G*_dec_. The corresponding spectra of the inverse filters in dB scale (right column) representing the attenuation or amplification effect for the values below or above the zero dB threshold (dashed horizontal lines), respectively. Dashed vertical lines show the valid frequency range of 10–350 Hz. Three selected sequences are shown with *.

The permutation of SOAs does not change the jitter ratio as defined in (Eq 8). However, the frequency properties, and consequently the NGFs of these permutated sequences are different. Fig 4 offers a direct presentation, using examples, of such a relationship between permuted sequences and Fourier representation of corresponding inverse filters. The left column contains 15 representative sequences arranged in an ascending order (Seq1–15) of *G*_dec_ values. The right column shows the sequence-dependent inverse filters in the range of 10–350 Hz in the Fourier domain. Table 1 provides more details about these 15 stimulus sequences, and their corresponding NGFs (*C*_dec_ and *G*_dec_). Note that these stimulus sequences have the same stimulation rate (39.1 Hz), jitter ratio (81%) and frequency range, while they differ in *C*_dec_ and *G*_dec_ values. The difference is also clear in the Fourier presentations of their corresponding inverse filters in Fig 4 (right column).

**Table 1:** Representative sequences and the corresponding NGFs.

Seq.ID	SOA arrangements	*C*_dec_	*G*_dec_
Seq1*	27.2; 36.8; 36.8; 20.8; 32.0; 19.2; 16.0; 16.0	0.52	0.68
Seq2	16.0; 16.0; 32.0; 36.8; 19.2; 27.2; 20.8; 36.8	0.72	0.74
Seq3	16.0; 16.0; 36.8; 20.8; 19.2; 32.0; 36.8; 27.2	2.62	1.19
Seq4	16.0; 19.2; 16.0; 20.8; 36.8; 32.0; 36.8; 27.2	3.01	1.60
Seq5	16.0; 19.2; 16.0; 36.8; 36.8; 20.8; 32.0; 27.2	0.71	1.91
Seq6*	16.0; 32.0; 16.0; 36.8; 36.8; 19.2; 20.8; 27.2	4.46	2.00
Seq7	16.0; 16.0; 19.2; 20.8; 32.0; 27.2; 36.8; 36.8	0.98	2.93
Seq8	16.0; 19.2; 27.2; 20.8; 16.0; 36.8; 32.0; 36.8	1.23	3.21
Seq9	16.0; 19.2; 20.8; 32.0; 36.8; 16.0; 36.8; 27.2	1.67	3.44
Seq10	16.0; 16.0; 20.8; 36.8; 32.0; 19.2; 27.2; 36.8	2.03	3.63
Seq11	16.0; 32.0; 19.2; 36.8; 16.0; 36.8; 20.8; 27.2	1.92	4.56
Seq12	16.0; 19.2; 16.0; 32.0; 36.8; 36.8; 27.2; 20.8	1.85	4.68
Seq13	16.0; 19.2; 16.0; 36.8; 36.8; 32.0; 20.8; 27.2	2.06	5.70
Seq14*	16.0; 16.0; 19.2; 36.8; 32.0; 20.8; 27.2; 36.8	2.10	5.93
Seq15	16.0; 16.0; 19.2; 36.8; 32.0; 20.8; 36.8; 27.2	1.93	6.52

* Three sequences are selected for the real CLAD paradigm recordings

### Correlations between NGFs and noise processing performance

We evaluated the goodness of a sequence for CLAD in terms of its noise amplification/attenuation property in the time domain during the deconvolution process. Quantitatively, we calculated the noise gain via measuring the relative output noise level after deconvolution in reference to the background EEG level before deconvolution. During the process of calculation, background EEG only were used as inputs to the deconvolution algorithm and actual noise gains (ANGs) were computed as the ratio of root mean square (RMS) voltages between post- and pre-deconvolved EEG:

$$ANG=20{\text{log}}_{10}\frac{\text{rms}(EEG_{\text{post}})}{\text{rms}(EEG_{\text{pre}})}.$$ (16)

When *ANG* < 0 dB, the inverse filter has noise attenuation property. The lower the ANG, the better sequence it is for CLAD. The ANG metric is a specific measurement for noise performance of a sequence on an instance of EEG data, so that it can be used to validate the NGFs.

**Fig 5:**
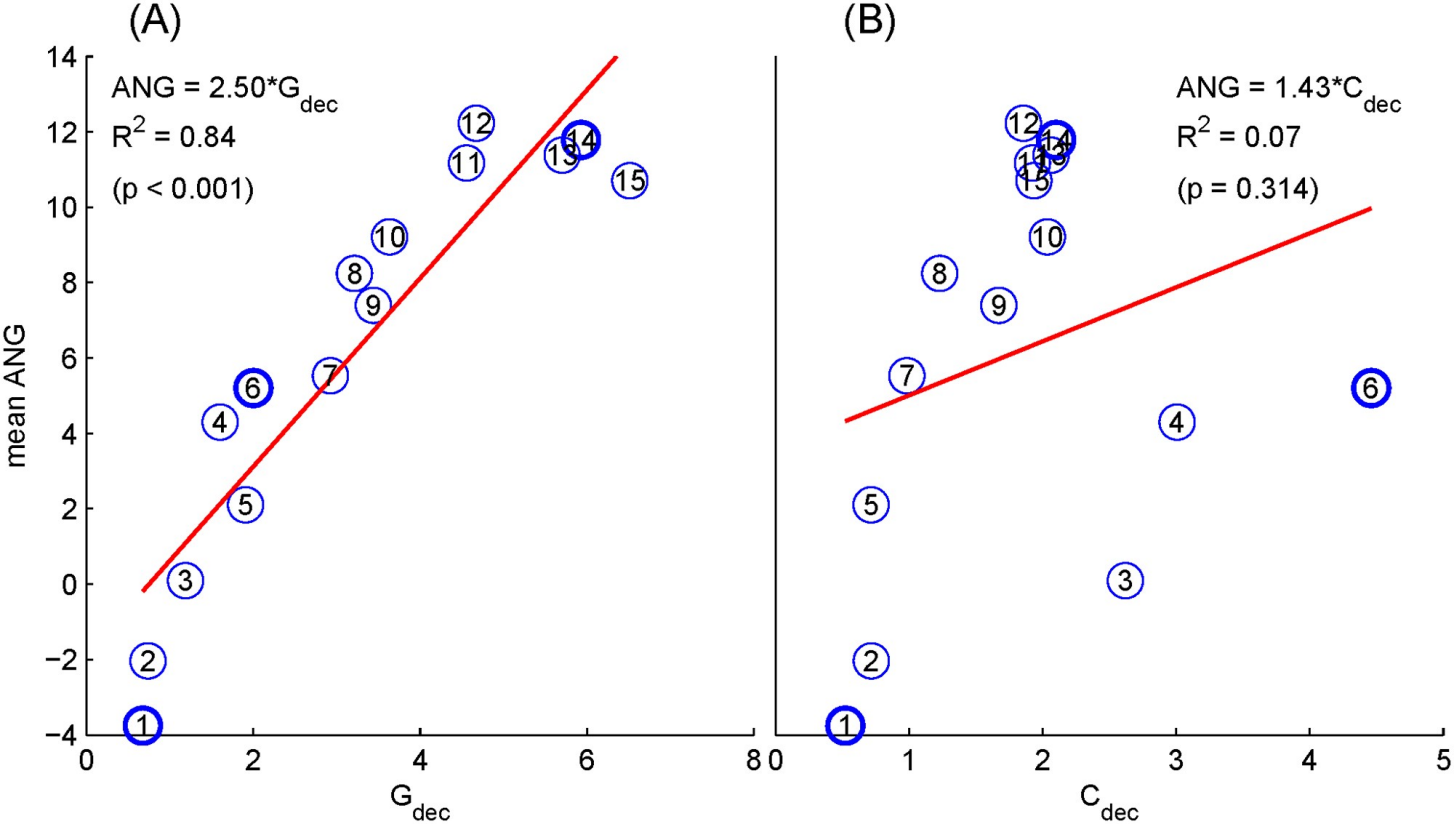
Linear regression between the NGFs measured by the values of *G*_dec_ (A) and *C*_dec_ (B), and the noise gains measured by the mean ANGs. The circled digits indicate the sequence index (Seq1-Seq15). Three selected sequences are shown in bold circles.

Since these sequences were designed for obtaining MLR, we selected EEG data randomly as in Fig 2B to evaluate their performance. We selected 150 epochs of raw EEG of 204.8 ms as a set of benchmark for testing. Fig 5 shows the linear regression between the averaged ANGs and NGFs. The numerical labels of these 15 sequences as in Table 1 are marked in the circles and, among them, three sequences used later in the CLAD testing are shown in bold circles. The linear regression lines are also presented. Ideally, a linear relationship is expected between NGFs and ANGs. A significant linear relationship can be observed using the proposed NGF measurement (*G*_dec_ in Fig 5A), and its regression coefficient and R-squared values are 2.50 and 0.84 (*p* < 0.001), respectively. However, significant relationship is not observed when using *C*_dec_, where the coefficient and R-squared values are 1.43 and 0.07 (*p* = 0.314), respectively (Fig 5B).

Sequences #6 (Seq6) and #14 (Seq14) were selected, as examples, to illustrate the cases with large inconsistencies between *C*_dec_ and ANG, as compared with the optimal case (Seq1), which has the lowest *C*_dec_, *G*_dec_, and ANG values and consistent *C*_dec_ and ANG values as well. The cases of Seq6 and Seq14 suggest different patterns in terms of the relationship between *C*_dec_ and ANG, where Seq6 has the worse *C*_dec_ (= 4.46), but moderate estimated ANG value while Seq14 shows the opposite condition. It is observed that the frequency distribution of the inverse filter of Seq6 (Fig 4) shows a large amplification at 260 Hz, which greatly increases values of *C*_dec_ since the *C*_dec_ metric considers the 260 Hz component equally as low-frequency components. However, it contributes less to the modified metric *G*_dec_ since *G*_dec_ takes the realistic 1/f distribution of EEG into the consideration. It is further noted that if the considered frequency band is wider, another high-frequency component peaked around 360 Hz might further deteriorate *C*_dec_, but not *G*_dec_. On the contrary, Seq14 shows only a large peak at the low frequency that should influence the deconvolving solution according to *G*_dec_ measurement, but was not pronounced in *C*_dec_. Finally, Seq1 has the optimal performance because amplification gains of all frequency components are below 1.

### Experiments of generating optimal sequences using Genetic Algorithm (GA)

Finding a CLAD sequence with minimal NGFs is a global optimization problem with no analytic solutions. The objective function of the optimization algorithm can be designed to minimize the NGF metric. Although it is generally desired to have the sequence with NGF as small as possible, some constraints on the sequence have to be considered. For example, stimulation rate and jitter ratio may be pre-determined by specific applications. We addressed this problem mathematically by a general global optimization approach that is subject to customized constraints.

In this experiment, we validated the superiority of the proposed NGF in generating appropriate CLAD sequences by means of a canonical genetic algorithm (GA) [27]. Other global optimization methods, such as simulated annealing, or differential evolution, are also applicable. The objective function for GA is

$$\phi (x)=NGF,NGF\in \{G_{\text{dec}},C_{\text{dec}}\},$$ (17)

where the variable represents a real vector of SOAs, and the dimension *P* represents the number of stimuli in a sweep that is given *a priori* according to specific applications. In this case, jitter ratio (JR) actually defines the constraint of possible sequences. For simplicity, we defined a box constraint with lower and upper bounds for ***x***, i.e., $x\in {\left[x_{\text{L}},x_{\text{U}}\right]}^{P}$, where *x*_L_ and *x*_U_ are the minimum and maximum of SOAs, respectively, so that the JR can be calculated using (Eq 8). Thus, the GA optimization is expressed in a nonlinear programming problem as

$${\text{min}}_{x}\{\phi (x)\},s.t.\begin{array}{c} {\begin{array}{cc} & x\in [x_{\text{L}},x_{\text{U}}] \end{array}}^{P}. \end{array}$$ (18)

**Fig 6:**
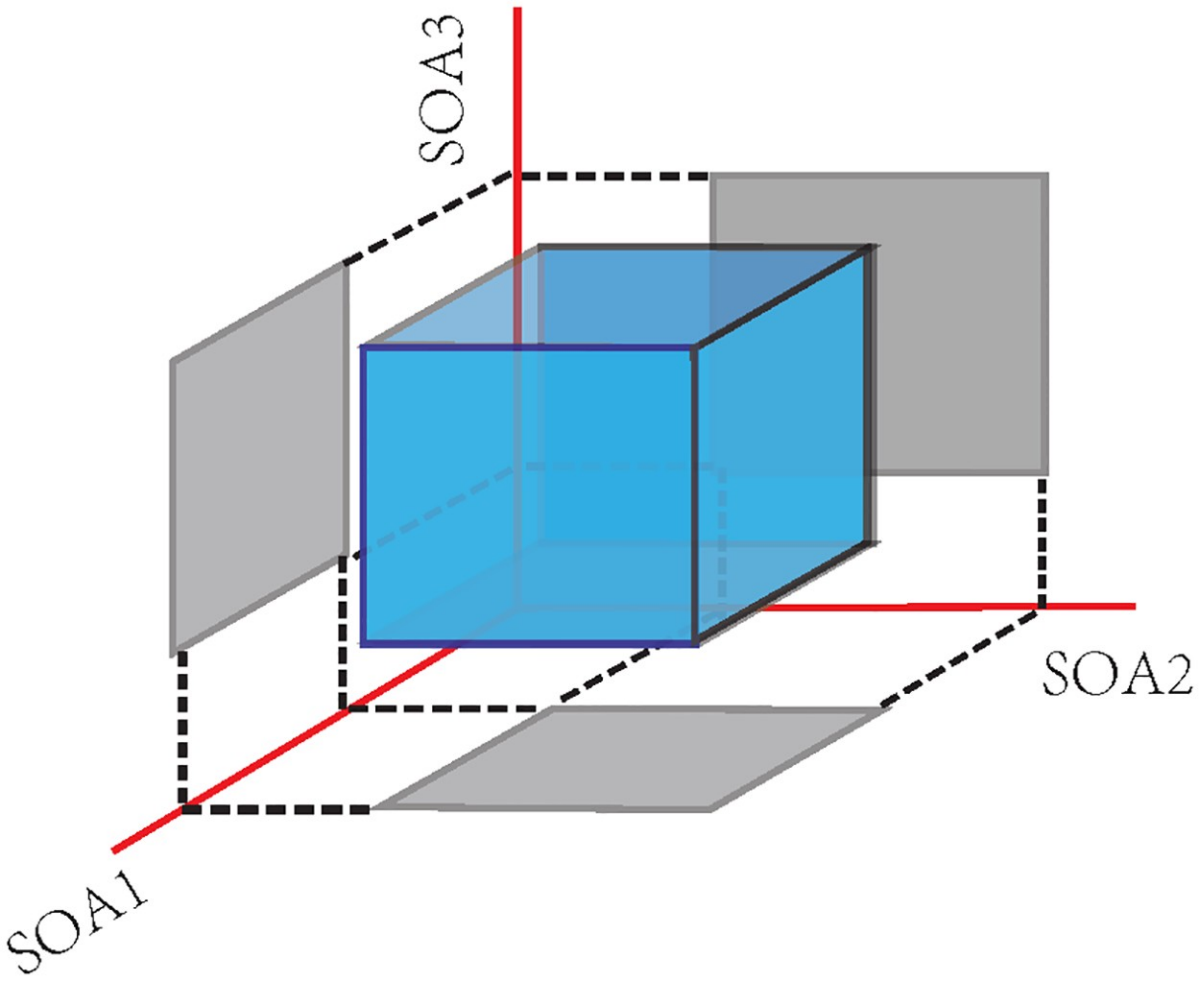
Diagram of GA searching space for three variables (*x*_1_, *x*_2_, and *x*_3_) which represent a stimulus sweep that contains three SOAs. Due to the constraint of JR, the searching space is confined within the cubic box.

The searching space for (18) is illustrated in Fig 6 as a cubic box in case of 3 dimensions (*P* = 3). Each dimension represents a SOA or an element of the vector ***x*** = [*x*_1_, *x*_2_, *x*_3_]. The constraint requires that the values of each element are in the range of *x*_L_ and *x*_U_, which is the interval projected onto the axes (Fig 6) of the cube. Optimization for more than 3 dimensions can be carried out in an analogous hypercube.

GA represents a typical nature-inspired metaheuristics, using mainly three operations in its evolution processes: mutation, selection, and crossover. And the constraint in (Eq 18) is handled by the penalty function approach [27]. In this experiment, we employed the ‘ga.m’ function from the MATLAB Global Optimization Toolbox (R2012b, the Mathworks, Inc., Natick, MA) for the implementation of the algorithm.

The number of stimuli *P* per sweep can be dilemmatic in the generation of CLAD sequences. Generally, smaller P is preferred because it is relatively easy to design a “good” CLAD sequence with fewer parameters to considered. Practically, a sweep with smaller P is shorter and, therefore, it saves EEG recording time. When it comes to the problem of rejecting noisy EEG data in the pre-processing preparation of useful EEG data, shorter sweeps mean less data sacrifice. However, smaller P values mean less number of jittered sequences with differently ordered SOAs, which reduces the chance in finding a “better” CLAD sequence with lower NGF values. Using the GA approach, we can offer an empirical relationship between the NGFs and *P*. We thus compared 20 trials with random initialization in GA iterations for *P*s from 4 to 11. Other settings for the sequence were similar with the MLR recording paradigms at a stimulation rate of about 40 Hz. The objective function was also defined on the same frequency band. The constraint bounds were 15 ms and 35 ms as in (Eq 18), therefore yielded 80% of JR accordingly. Note that the constraint could not guarantee fixed length of a sweep, and the exact values of stimulation rate and JR might vary around the designated values.

**Fig 7:**
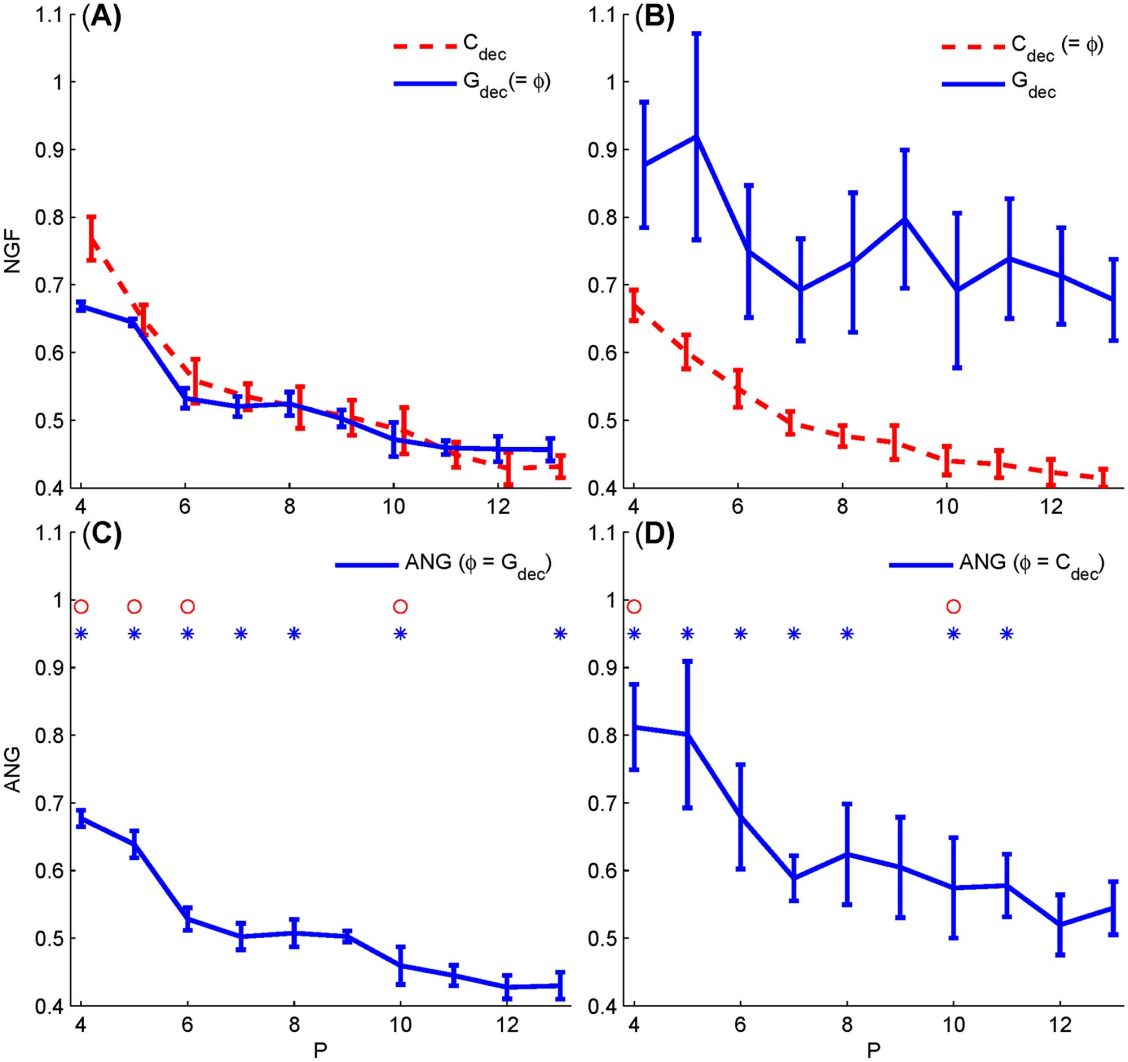
NGFs and ANGs for the optimized sequences with respect to the stimulus number from 4 to 13 in a sweep. Both *G*_dec_ (solid) and *C*_dec_ (dashed) values are plotted for sequences optimized based on objective function of *ϕ* = *G*_dec_ (A) and *ϕ* = *C*_dec_ (B), respectively. The corresponding ANGs for these sequences based on *G*_dec_ and *C*_dec_ are presented in (C) and (D), respectively. The symbols ‘*’ and ‘o’ indicate the cases with statistical significance for *G*_dec_, and *C*_dec_, respectively.

Since the solution of GA was sensitive to the initial state, we thus carried out 11 runs with random initialization to avoid biased results. The mean (± std) NGFs with respect to *P*s from 4 to 11 optimized by the use of either *G*_dec_ or *C*_dec_ in the objective function are shown in Fig 7(A) and 7(B), respectively. It shows that the NGFs of *G*_dec_-optimized sequences decline with the increased *P*s (solid trace in Fig 7A), and the corresponding *C*_dec_ values (dashed trace in Fig 7A) of these sequences are consistent with *G*_dec_ as well, suggesting that sequences with small *G*_dec_ values are likely to have small *C*_dec_ values that can be also observed in Fig 3B. In contrast, such an association does not exist if the sequences are optimized by the *C*_dec_ metric (dashed trace in Fig 7B), in which the corresponding *G*_dec_ values (solid trace) are evidently worse (larger value, and variation) for all *P*s.

Since the generated sequences were different in terms of two NGFs, as indicated in Fig 7, we might expect that the *G*_dec_ can present a better measurement of noise attenuation property in practice. We could further justify this notion in terms of ANG as in (Eq 16) via processing real EEG with the deconvolution filters. Specifically, for an optimized sequence, we filtered 15 stimulus-free EEG epochs to yield an averaged ANG. These ANGs corresponding to sequences optimized by *G*_dec_ and *C*_dec_ are shown in Fig 7C and 7D, respectively. Basically, the ANGs are decreased with the length of sequence (indicated by *P* in the horizontal axis) following the NGF pattern. This is particularly true in the case of *ϕ* = *G*_dec_ (Fig 7A and 7C). But it can be observed that ANGs based on *C*_dec_ (Fig 7D) is more closely related to *G*_dec_ rather than *C*_dec_ (Fig 7B) by the facts that both of them exhibit elevated values and increased variation. We quantified the relationship of ANG and two NGFs on the optimized sequences for the same *P* as shown in Fig 7C and 7D in which the symbols ‘*’ and ‘o’ indicate the correlation with significance (*p* < 0.05) for cases of *G*_dec_ and *C*_dec_, respectively. It shows that the ANG is more likely to correlate with *G*_dec_ than *C*_dec_. These results are in agreement with the established relationship between NGFs and ANG (c.f. Fig 5), which further confirms the merit of the *G*_dec_ metric.

The decline of NGF (and ANG) with increased *P* suggests a preference for more number of stimuli in a sweep. However, such a merit is priced at long sweep length or recording duration. A fair measurement for noise attenuation may be reasonably defined as the noise gain over the same length of recording. In general, the whole data processing for the CLAD paradigms includes first the ensemble averaging over a sweep of raw EEG (looped average) and then the deconvolution filtering on averaged responses. Both steps impact noise properties. As is known that the signal-to-noise (SNR) improvement for *K* times average is by a factor of [28], the overall NGF including the looped-average can be expressed as

$$NGF_{\text{tot}}=\frac{1}{\sqrt{k}}NGF.$$ (19)

Suppose that the mean SOA is the same for all sequences, the total length of stimulus sequences is *K*·*P*·SOA, indicating that *K* is in proportion to 1/*P* in order to have similar recording time (sequence length). By this relationship, (Eq 19) becomes

$$NGF_{\text{tot}}\propto \sqrt{P}\cdot NGF$$ (20)

where $\sqrt{P}$ is used as an adjusting factor when noise attenuation from ensemble averaging is being included.

**Fig 8:**
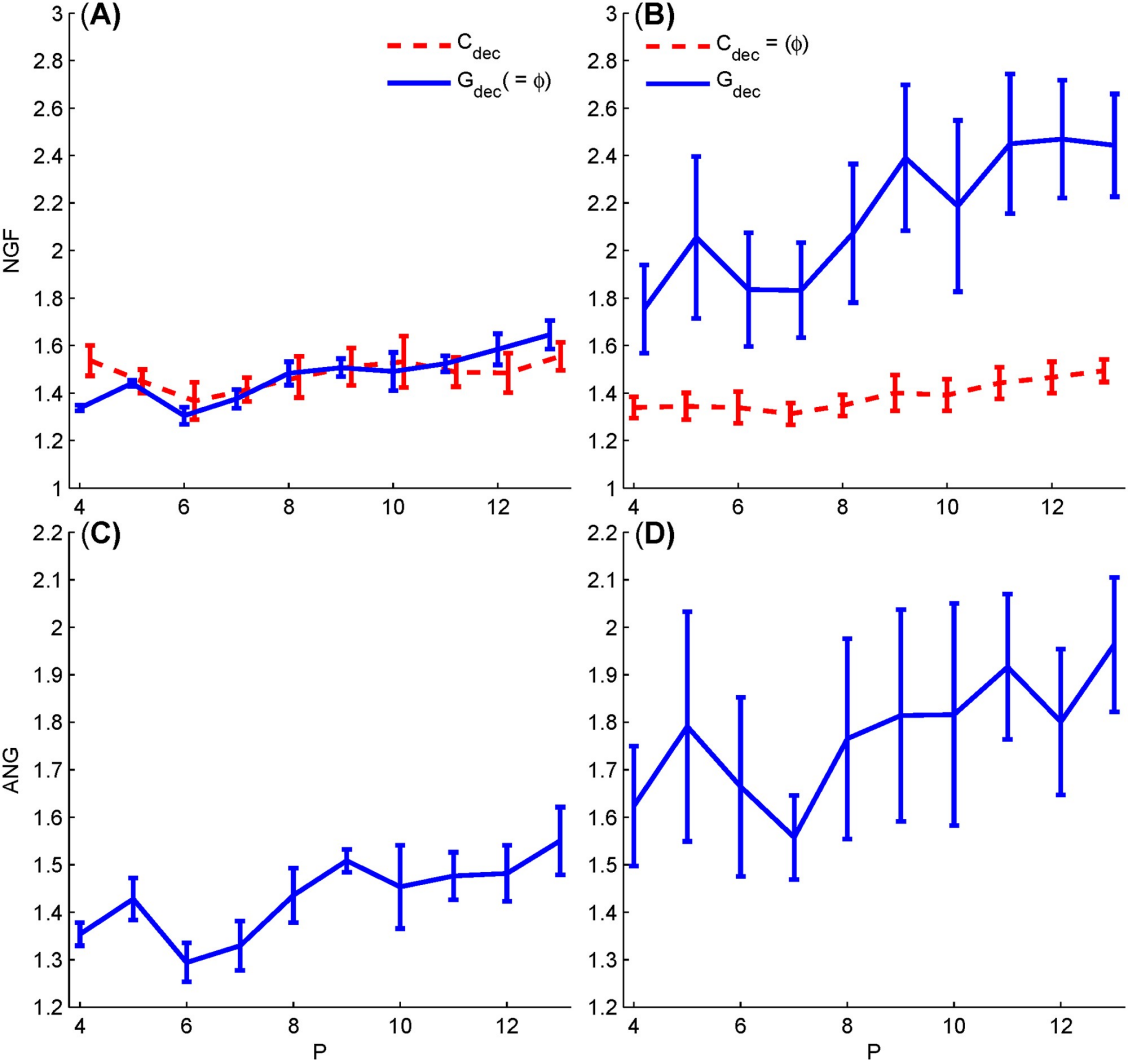
Adjusted NGFs and ANGs for the optimized sequences with respect to the stimulus number from 4 to 13 in a sweep. Both *G*_dec_ (solid) and *C*_dec_ (dashed) values are plotted for sequences optimized based on objective function of *G*_dec_ (A) and *C*_dec_ (B), respectively. The corresponding ANGs for these sequences based on *G*_dec_ and *C*_dec_ are presented in (C) and (D), respectively.

We therefore recalculated the NGFs and ANGs as in Fig 7 with this adjustment factor $\sqrt{P}$, and illustrated the results in Fig 8. The adjusted NGFs and ANGs reverse the decline trend to an overall uplift with increased *P*s. This counteractive effect of *P* on NGFs gives rise to a problem on the selection of number of stimulus in a sweep. The uprising pattern of ANG in Fig 8 might suggest that short sweep length is favored in terms of overall SNR. However, we cannot firmly sustain this conclusion without further comprehensive investigations, because GA cannot guarantee the convergence to the global optimization solution, in particular the optimizing process is more difficult for larger searching dimension as *P* increases.

### Comparison of 40-Hz transient AEP obtained from selected sequences

The results shown in Figs 5 and 7 indicate that the *G*_dec_ metric is more correlated to noise attenuation performance than *C*_dec_, indicating the merit of the new NGF in measuring the noise gain property by comparing noise levels on stimulus-free EEGs. This experiment was performed to compare deconvolved AEPs using three sequences from Table 1 (Seq-1, 6 and 14). These sequences featured with different NGF measures. Seq1 is an optimal sequence with the smallest *G*_dec_ and *C*_dec_. Seq6 ranks the sixth in performance among the 15 sequences by *G*_dec_ (= 2.00), but is the worst sequence by *C*_dec_ (= 4.46). Seq14 is a representative "bad" sequence as measured by *G*_dec_ (= 5.93), but a moderate sequence by *C*_dec_ (= 2.10).

**Fig 9:**
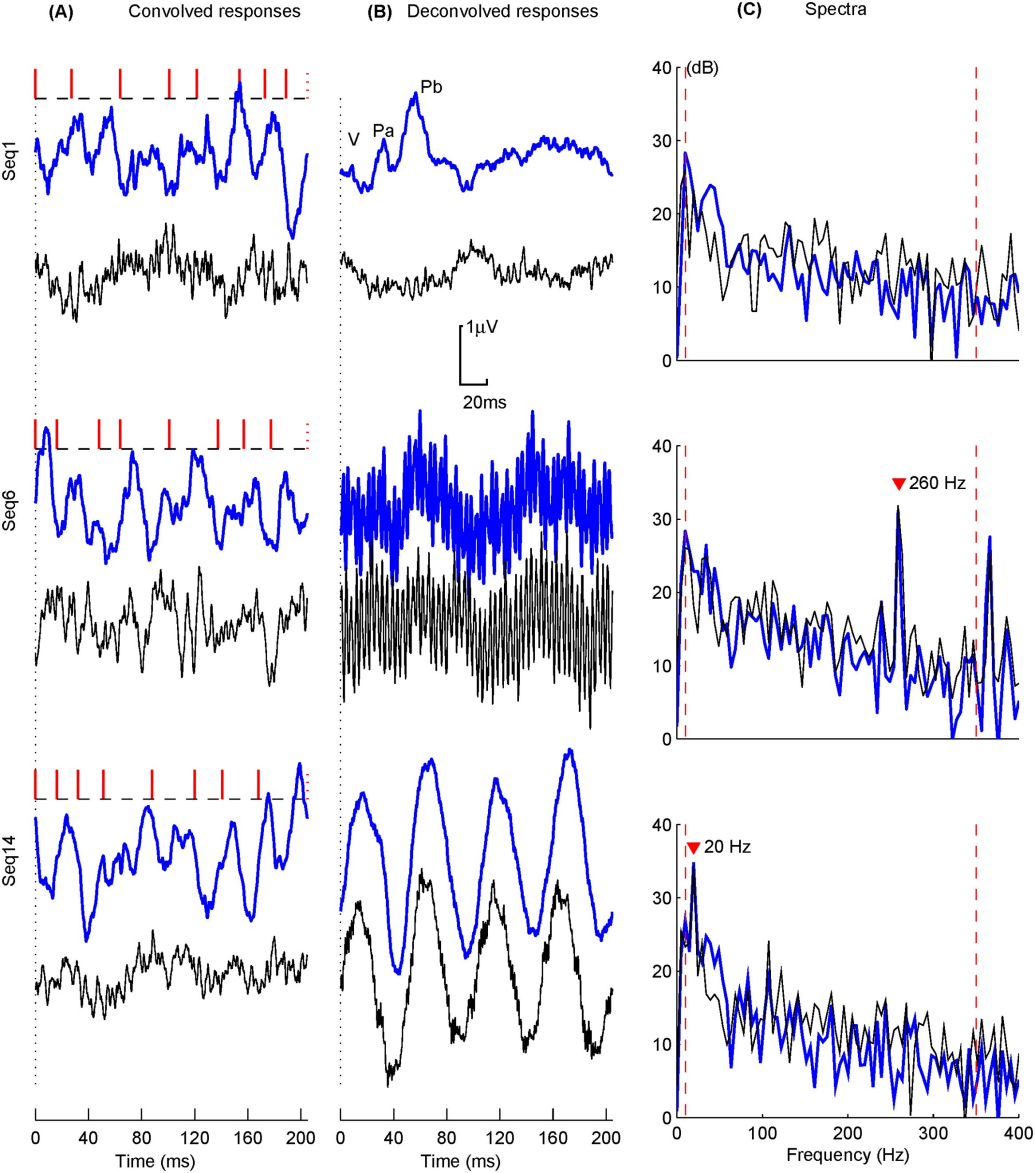
Auditory responses and corresponding spectra for pre- and post- deconvolution for a typical subject (Sub1) using the selected sequences (Seq1, Seq6 and Seq14). Thick and thin traces indicate the estimated responses and noises, respectively. The filled "∇" markers indicate the primary peaks in the spectral plots (C) causing the oscillations seen in the 2nd column (B).

Responses from a typical subject (Sub1) are shown in Fig 9. The convolved responses averaged over 1800 EEG sweeps (thick traces), and the estimated noise (thin traces below) are shown in Fig 9A. The noise was estimated by ± reference method obtained by the subtraction of responses from even- and odd-numerated EEG sweeps [29]. The corresponding deconvolved transient AEPs (thick traces) and filtered noises (thin traces) are shown in Fig 9B. Fig 9C shows the spectra of the estimated AEPs and noises.

It can be observed that the convolved responses and noise waves of these three sequences demonstrate no evident SNR differences (Fig 9A). The deconvolved responses of these three sequences, however, exhibit quite different profiles. The AEP for Seq1 is typical in accordance with previous reported results using the same sequence [26]. The ABR component, wave-V, and the main MLR components, namely, P_a_ and P_b_ are obviously recovered as labeled. The corresponding frequency distributions of AEPs and filtered noise signals demonstrate a typical frequency property with more pronounced components in the low frequency range.

The ABR and MLR components for Seq6 are hardly recovered in Fig 9B, since a significant amount of high frequency interference noises are present. The corresponding spectrum shows two high frequency components corresponding to the frequency characteristics of the inverse filter for Seq6 (see Fig 4). In spite of heavy high frequency distortions, the main AEP profile is still observable. This property is consistent with the *G*_dec_ value of this sequence rather than its *C*_dec_ value.

The AEP response for Seq14, however, is hardly acceptable since a dominant low frequency oscillation overrides the main Pa and Pb components of MLR. This result is useless because the abnormally amplified frequency component within the AEP’s main frequency range impacts the response signal entirely. The corresponding spectrum shows an over-amplified component at approximately 20 Hz because of the improper frequency characteristic of the sequence. The low value of *C*_dec_ fails to properly measure the noise performance of this sequence by not giving a high weight toward low frequency noise amplification as displayed in Fig 4.

**Fig 10:**
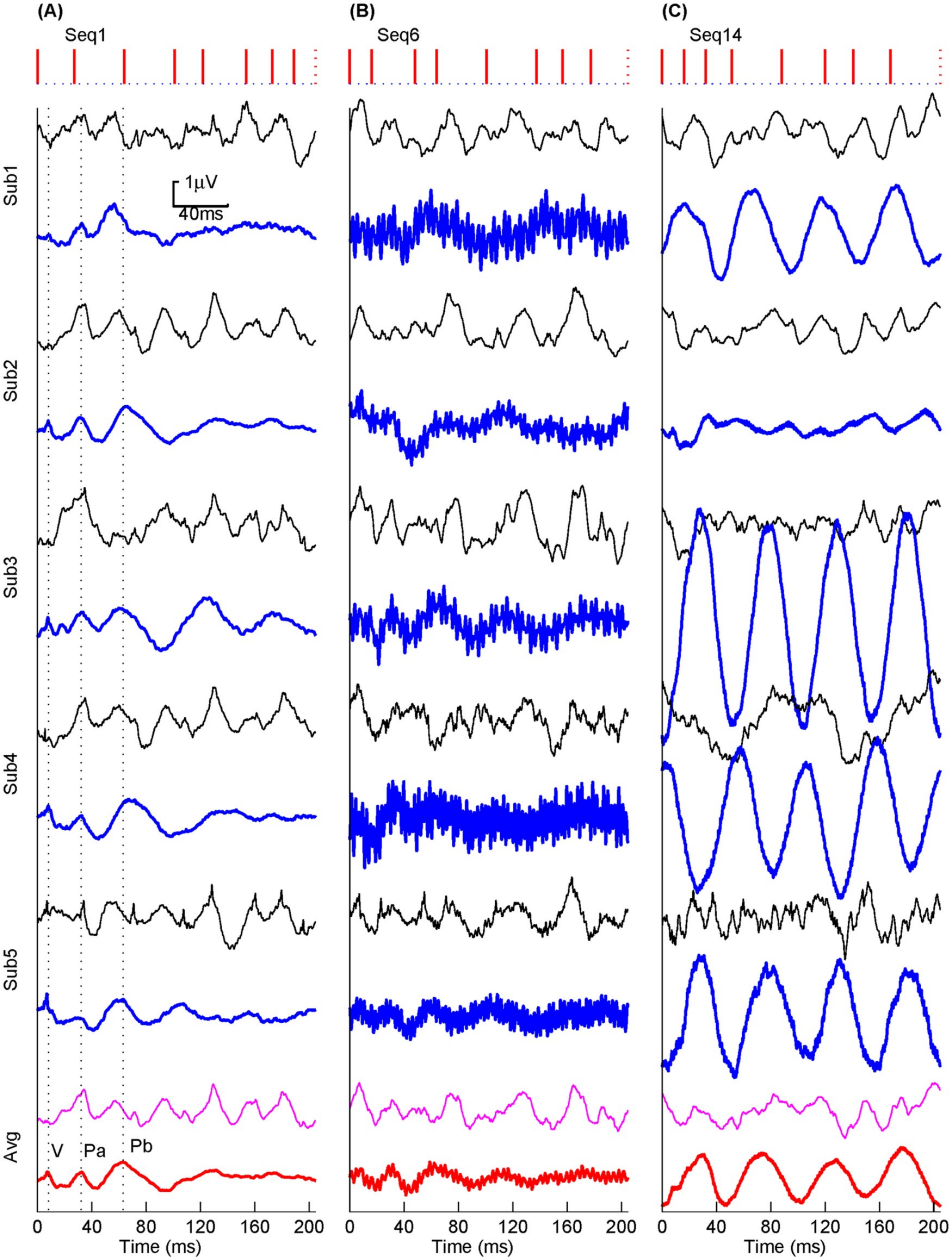
Pre- (thin traces) and post-deconvolution (bold traces) responses from five subjects (Sub 1–5) and the grand averages over them (Avg) for the three representative sequences (Seq-1, 6 and 14, in columns A, B and C, respectively).

Fig 10 shows the deconvolved AEPs of these sequences for all subjects and the grand averages, respectively. The consistent AEP profiles indicated by the NGF properties are observed for all subjects. Except for AEPs by Seq1, the main components are hardly identifiable for individual AEPs by other sequences. After averaging over five subjects, the resulting AEP waveform is largely improved and well-defined for Seq6 in which main ABR-MLR components can be identified, while it is still severely distorted for Seq14. This is because that high-frequency noises are easier to be canceled than low-frequency noises by averaging. These results also justify the improvement of the proposed metric in measuring the sequence property.

These results from five subjects are only the examples to demonstrate how the sequences would affect the deconvolved responses and how the proposed metric can be more appropriate to measure the ‘goodness’ of these sequences. In practice, it is advised to verify the noise property to guarantee the fitness of 1/*f* model.

## Discussion

The CLAD method has been proved to be an efficient technique in the study of evoked responses under rapid stimulation paradigms. Designing or generating appropriate sequences is a prerequisite in the application of CLAD. The CLAD paradigm employs more stimuli than the conventional low rate paradigm given the same recording time. However, the transient AEP obtained from the deconvolution process does not necessarily improve SNR if the frequency property of the inverse filter sequences is not well-tuned. Although efforts have been made aiming to alleviate the noise amplification issue using time or frequency domain filters [23,30], a fundamental solution would be of obtaining an optimal stimulus sequence with desired noise gain property that requires a metric to gauge the performance of a sequence for specific applications.

In the present study, we developed a new NGF metric, *G*_dec_, that effectively quantifies the sequence property based on the consideration of the pattern in EEG noise characteristics. Considering the ubiquitous nature of the 1/*f* random process, the basic idea of developing *G*_dec_ is to incorporate noise pattern into the weight function design of the deconvolution filter. This new metric is justified in typical EEG data and demonstrated to be both necessary and adequate in characterizing noise property of a sequence, which makes it appropriate as an objective function for obtaining optimal sequences.

Basically, there are two parameters used in the *G*_dec_ metric in practice. The exponent α value controls the damping degree of noise magnitude with increased frequency. It can be readily estimated by least squares fitting on frequency characteristics of spontaneous EEGs. *G*_dec_ is equivalent to the original NGF metric, *i*.*e*., *C*_dec_, for α = 0. Our analysis on sufficient large sets of EEG data indicates that the α value is close to 1, which makes spontaneous EEG spectrum a standard 1/*f* distribution.

Another parameter that impact original *C*_dec_ values is the considered frequency range. In practice, the low-cutoff frequency *f*_L_, is important and relatively fixed according to the AEP components to be investigated, while the high-cutoff frequency can be varied in a wide dynamic range. Since *G*_dec_ is more sensitive to low-frequency components, selecting the *f*_L_ is critical in a searching algorithm. Lower *f*_L_ may affect the converging speed during iterations for an optimization algorithm. Even equipped with necessary filters from acquisition systems, we recommend applying an offline bandpass or essentially highpass filter again before the deconvolution calculation in case signal energy outside investigated frequency bands can be reintroduced in the averaging process before deconvolution. In contrast, the selection of high-cutoff frequency can be widely varied in practice. The *G*_dec_ is advantageous over the original *C*_dec_ metric in the sense of its stability over a variable high frequency range, since *G*_dec_ is naturally less sensitive to high frequencies according to its weighting strategy.

As indicated in the derivation of (Eq 11), the length of sequence sweeps determines discrete frequency interval in the frequency domain. For example, the frequency resolution for the initial sequence of the present study is 4.88 Hz for the sweep length of 204.8 ms, implying that the first three values of *F*(*k*Δ*f*) represent 0–9.76 Hz frequency bins that are outside the low range of frequency bandpass filter (10 Hz). Increasing the sweep length that contains more stimuli is theoretically allowed for the fine control of frequency distribution, but it complicates the selection or optimization procedure of sequences.

Sequence optimization is a critical problem that has not been fully studied which hinders scientific and clinic applications of the CLAD method. The present study shows that a ‘good’ sequence appears to be associated with a small original metric *C*_dec_, whereas a small *C*_dec_ is not always associated with a good sequence. Thus, this metric is only necessary but not adequate in finding optimized sequences, so that it is not appropriate to be incorporated in the objective function to be solved by an optimization algorithm. The new metric *G*_dec_ developed in the present study has demonstrated a satisfactory linear relation to noise attenuation property that makes it a better candidate to be incorporated in an objective function. Thereafter, we are capable of designing CLAD sequences with guaranteed performance using a global optimization strategy.

The *G*_dec_ defined in time-continuous version make it particularly suitable in many random searching optimization algorithms in real number field. Moreover, it may also benefit from the continuous searching space that facilitates the finding of optimal sequences. As illustrated in Fig 6 that the solution is within the hypercube space due to the JR constraint, considering the opposite effect of JR and NGF on optimization, we can conjecture that optimal solutions may finally converge to the surface of the hypercube. Therefore, more pertinent modification on the optimization algorithm can be implemented for the future work.

An increasing number of studies using CLAD were reported in the literature and, therefore, the noise property of CLAD sequences has to be examined to guarantee reliable deconvolution performance. Some applicable sequences were presented for the convenience of similar uses in the study of MLR components [16,18, 26,31]. These sequences have been evaluated in terms of *C*_dec_ values or by visual inspection on their spectra. In addition to initial applications in ABR and/or MLR, the CLAD method was recently successfully used in other modalities. For example, CLAD sequences have been used to derive transient patterns in electroretinogram data at a rate from a large range, in which both *C*_dec_ and sequence spectra were given [25,32]. More recently, a combination of Electrocochleography/ABR data were acquired at rapid rates using six CLAD sequences with appropriate *C*_dec_ values [20]. To date, no report on generating optimal CLAD sequences is available based on a NGF metric. As indicated in the present study, the proposed *G*_dec_ can be better correlated to actual noise attenuation property, which is promising in optimizing or generating CLAD sequences with desired properties.

Besides CLAD, a few other deconvolution methods based on the superimposition hypothesis [5,6] were proposed. These methods should be used with caution for the reliability of deconvolution. For the well-established MLS method [7], the deconvolving process is transformed into a cross-correlation calculation without inversion operation, so that no noise amplification would occur. As it was measured by *C*_dec_ [11], which treated MLS as a subset of CLAD sequences, MLS was proved to be an optimal CLAD sequence. A recent deconvolution method proposed by Valderrama et al [13] employed only time averaging operation based on sequences with randomized SOAs to cancel out unwanted components. This method thus did not suffer noise amplification issue at a price of imposing adequate jitters [13]. A different jittering scheme proposed by Wang et al [15] utilized matrix inverse in deconvolution, and regularization techniques were introduce to stabilize solutions under influence of noise. In summary, errors arising from deconvolution techniques should be fully investigated in the presence of noise.

## Conclusion

The present study is a pragmatic attempt to address the sequence generation and evaluation issues, which impede the acceptability of the CLAD method for clinical applications. Since noise gain property of the CLAD method can be characterized by frequency patterns of both stimulus sequence and background noise, appropriate modeling of background noise has a large effect on obtaining reliable solutions for deconvolution problems. Sequences with similar SOAs arrangements might exhibit dramatic variations that fail to present veridical AEPs. The proposed metric on NGF deliberately incorporates spectral characteristics of background EEG noise in an effort to significantly improve the measurement accuracy on NGF. In addition, the new metric is derived from a time continuous version which makes it more appropriate and convenient in the use of global optimization tools. The conspicuous effects indicated in both experimental and simulated data have showcased the efficacy of sequences obtained through the use of the new metric.

## Appendix

The following MATLAB (R2012, MathWorks, Nantik, MA) code can be used to calculate *G*_dec_, in which three variables need to be defined as input arguments. ‘soams’ is a vector containing the SOA values in ms for a CLAD sequence; ‘fL’ and ‘fH’ are low and high cutoff frequency values; alpha is a variable in the 1/f^α^ model that can be set to 1 for most EEG recordings.

---------------------------------------------------

function gdec = ngf(soams, fL, fH, alpha)

% ---- input---------

% soams: vector of clad sequence SOA in ms.

% fL and fH: low and high valid frequency band.

% alpha: usually = 1 in the 1/f^alpha EEG model

% ---- output----

% gdec: noise gain ratio for the proposed metric

%---------------------------------------------------

soa = soams/1000; % transfer SOA unit into second

tk0 = cumsum(soa); %

T = tk0(end); % length of the CLAD sequence

tk = [0,tk0(1:end-1)]; % stimuli timing in a sequence

f0 = 1./T; % frequency resolution

w0 = 2*pi./T;

n1 = round(fL./f0) +1; % discrete low and high valid frequency

n2 = round(fH./f0)+1;

n = n1: n2;

fk = exp(-1i.*(w0.*n')*tk(1:end)); % Fourier transform

ftk = sum(fk,2);

H = 1./(ftk.*conj(ftk)); % inverse filter

f_new = 1./(n'.*f0).^(2*alpha);

gdec = sqrt(sum(H.*f_new)/sum(f_new));

----------------------------------------------------------------------

The major MATLAB code for generating the optimal CLAD sequence is to use the ‘ga’ function provided by the ‘Global Optimization Toolbox’. The predefined variables for this code is the number of SOA in a sequence, and two arguments of ‘LB’ and ‘UB’ that define the low boundary for the initial minimum SOA, and the upper boundary for the initial maximum SOA. Both of them can be estimated from the jitter ratio defined in (Eq 8) use the unit of millisecond. The *G*_dec_ function, i.e., ‘ngf.m’, can be modified to be the fitness function, i.e. the objective function ϕ defined in (Eq 17), used in ‘ga’ by keeping only one input argument ‘soams’ and assigning the rest arguments inside the function. Then use the code:

[soa,fval] = ga(@ngf,n,[],[],[],[],LB,UB,[]); to produce a number of optimized SOAs in the first output variable ‘soa’, and the corresponding fitness function values in ‘fval’. This is a random searching mode for GA method, and the result can be different for each run. We recommend multiple running to have the best sequence.

## Supporting information

S1 File Zip-compressed EEG data file. (ZIP)
